# Comprehensive biomarker profiling reveals distinct molecular signatures across stone types: a large-scale cross-sectional study in Southern China

**DOI:** 10.3389/fphys.2025.1612585

**Published:** 2025-06-09

**Authors:** Qingjiang Chen, Linliang Huang, Suilin Wang, Daqiang Wei, Jiancai Lu, Xiujing Han, Zhenglin Chang

**Affiliations:** ^1^ Department of Urology, Yuebei People’s Hospital Affiliated to Shantou University Medical College, Shaoguan, China; ^2^ Department of Urology and Guangdong Key Laboratory of Urology, The First Affiliated Hospital of Guangzhou Medical University, Guangzhou, China; ^3^ Department of Clinical Laboratory, State Key Laboratory of Respiratory Disease, National Center for Respiratory Medicine, National Clinical Research Center for Respiratory Disease, Guangzhou Institute of Respiratory Health, The First Affiliated Hospital of Guangzhou Medical University, Guangzhou, China; ^4^ Department of Orthopedics, Guangzhou Orthopedic Hospital, Guangzhou, Guangdong, China

**Keywords:** stone disease, urinary calculi, biliary calculi, serum creatinine, cystatin C, PSA, monocytes, β2microglobulin

## Abstract

**Background:**

Stone diseases represent a significant global health burden affecting 10%–15% of the population worldwide. Despite advances in diagnostic imaging, current approaches often lack the ability to predict stone formation or differentiate between stone types at early stages.

**Methods:**

This retrospective study analyzed data from 61,310 stone patients and 55,010 matched controls using 1:1 propensity score matching. Stone cases were categorized into five major groups and further subdivided by organ system. Comprehensive serum biomarker profiling was conducted using automated biochemistry analyzers.

**Results:**

Urinary system stones constituted the largest proportion (80.97%), followed by biliary system stones (21.12%). The study revealed distinct biomarker signatures: elevated serum creatinine and cystatin C in uric acid stones; increased PSA and monocyte counts in prostatic calculi; elevated β2-microglobulin and total bilirubin in common bile duct stones; and increased basophils, ceruloplasmin, ferritin, immunoglobulin-A, and rheumatoid factor in gallstones.

**Conclusion:**

This study represents the first comprehensive evaluation of stone-specific clinical biomarker patterns derived from routine laboratory parameters, providing potential diagnostic markers for different stone types and suggesting stone-specific pathophysiological mechanisms.

## Introduction

Stone diseases, including urinary and biliary calculi, represent a significant global health burden affecting 10%–15% of the population worldwide, with increasing prevalence across different demographics (Chewcharat and Curhan, 2021; Wang et al., 2023). The economic impact is substantial, with annual healthcare costs exceeding $10 billion in developed countries (Sturgis et al., 2022). Despite advancements in diagnostic imaging techniques like computed tomography and ultrasonography, current approaches often lack the ability to predict stone formation or differentiate between stone types at early stages. While traditional biochemical markers have been utilized in stone disease diagnostics, their clinical utility has been limited by insufficient specificity and the absence of comprehensive profiling across different stone types (Brisbane et al., 2016; Hanqi et al., 2019). Several studies have demonstrated that specific urinary markers can indicate stone risk, but these findings have not been systematically validated across large populations or different stone types (Brewin et al., 2021).

Despite significant advancements in molecular biology research in recent years that have greatly enhanced our understanding of the pathogenesis of stone diseases, the existing literature exhibits three critical limitations. First, most studies have focused on single organ systems (e.g., urinary tract or biliary tract stones) (Tamborino et al., 2024; Peerapen and Thongboonkerd, 2023; Peerapen and Thongboonkerd, 2021), with a notable absence of comparative analyses across different stone types. This oversight prevents the identification of shared molecular pathways or disease-specific biomarkers among distinct stone-forming conditions. Second, current biomarker research predominantly relies on high-cost omics technologies (e.g., proteomics or metabolomics) (Gao et al., 2022; Thongprayoon et al., 2022; Ma et al., 2024), rendering these findings difficult to translate into routine clinical testing. Furthermore, systematic validation using large-scale conventional laboratory parameters remains lacking. Third, prior attempts at cross-system analyses (Jing et al., 2024) have been limited by small sample sizes (n < 1,000) and heterogeneous control group designs, failing to adequately account for confounding factors such as age and gender, thereby casting doubt on the reliability of their conclusions.

In this study, we conducted a comprehensive analysis of 61,310 stone cases and 55,010 matched controls to identify distinct biomarker signatures across five major stone categories and their subtypes. By employing propensity score matching and advanced statistical approaches, we aimed to establish stone-specific molecular profiles and evaluate their diagnostic potential. Our analysis encompassed both traditional biochemical markers and novel molecular indicators, seeking to uncover unique patterns that could enhance our understanding of stone pathogenesis and improve diagnostic strategies. This study represents the largest systematic evaluation of biomarker profiles across different stone types to date, with the potential to establish new paradigms for stone disease diagnosis and management.

## Methods

### Study design

This retrospective study analyzed data from 61,310 stone patients diagnosed at the First Affiliated Hospital of Guangzhou Medical University, a single center, between June 2013 and June 2023. The stone data were first categorized into five major groups based on the location and type of the stones. These groups included Urinary Calculi, Biliary Calculi, Gout, Otoconia, and Miscellaneous Stones. The “other stones” category encompassed hepatic stones, testicular stones, gastric gland stones, submandibular gland stones, conjunctival stones, seminal vesicle stones, spermatic cord stones, and gastric stones. Subsequently, Urinary Calculi were further subdivided into five specific types: kidney stones, ureteral stones, bladder stones, urethral stones, and prostatic stones. Similarly, Biliary Calculi were classified into three specific types: gallstones, gallbladder stones, and bile duct stones. This hierarchical classification method facilitates the detailed analysis of system-specific and metastasis-associated biomarkers, aiding in the identification of organ-specific biomarkers. To establish an appropriate control group, we collected health check-up data from 55,010 stone-free individuals. A 1:1 propensity score matching was performed for each stone type to control for age and gender distribution, minimizing demographic bias. Each participant underwent comprehensive clinical laboratory tests, with each test having at least 50 valid measurements, and each individual having at least 10 test items. This multi-step approach enables the identification of both local and systemic biomarkers, providing deeper insights into stone-specific molecular characteristics. Statistical significance was assessed using t-tests, and biomarker changes were quantified using log2 fold-change calculations. This retrospective study was conducted in accordance with the Declaration of Helsinki and its later amendments. This study was approved by the Institutional Review Board at the First Affiliated Hospital of Guangzhou Medical University (Approval Number: ES-2025-K062). The requirement for informed consent was waived by the Ethics Committee of the Institutional Review Board at the First Affiliated Hospital of Guangzhou Medical University as this retrospective study analyzed only de-identified existing clinical data without patient contact, presented minimal risk to participants, and obtaining consent from our large cohort would have been impracticable. All patient information was fully anonymized to protect privacy throughout the research process.

### Blood sample examination

Blood samples were centrifuged at 3,000 rpm to separate the serum, which was then collected for analysis using an automated biochemistry analyzer, as previously described (Chang et al., 2022). This process yielded data on 96 different serum indicators. In the case of stone patients, serum samples were collected either during their hospital stay or at discharge. All testing was performed by the Clinical Laboratory Department of the First Affiliated Hospital of Guangzhou Medical University.

### Propensity Score Matching

Propensity Score Matching (PSM) is a statistical technique used to address selection bias in observational studies by estimating a propensity score for each participant (Chang et al., 2024). This score is then used to pair individuals from different groups, thereby controlling for potential confounding factors and improving the comparability between the groups. In this study, age and gender were chosen as covariates for matching, with a 1:1 ratio between patients and healthy controls, where each patient was matched with two healthy individuals who had undergone medical check-ups. To assess the quality of the matching process and the validity of the results, balance tests were conducted post-matching. Standardized mean differences (SMD) were calculated and maintained below 0.05, indicating that the matching procedure effectively balanced the covariates between the two groups.

### Differential analysis

Differential analysis is used to identify significant differences between groups, helping to assess the impact of specific factors or variables. It provides statistical significance (p-value) through hypothesis testing, ensuring that observed differences are meaningful and not due to random variation, thereby supporting decision-making and inference. In this study, statistical significance (p-value) and effect size (log-transformed fold change) were used to select differential indicators, applying a p-value threshold of 0.05 and a fold change of 1.5.

### Receiver Operating Characteristic

The Receiver Operating Characteristic (ROC) curve is a graphical tool used to evaluate the diagnostic performance of a binary classification model, illustrating the trade-off between sensitivity and specificity at various thresholdsThe area under the curve (AUC) quantifies the overall ability of the model to distinguish between positive and negative cases, with a higher AUC indicating better diagnostic accuracy. In this study, an AUC greater than 0.70 was set as the threshold to select biomarkers with high diagnostic potential.

### Statistical analyses

All statistical analyses were performed using R (version 4.0.2) and IBM SPSS Statistics (version 25.0). Differential analysis was carried out using the ‘limma’ package in R. Chi-square tests were applied to examine differences in categorical variables, which were presented as percentages (%). For continuous variables, statistical significance between two groups was assessed using either the Student’s T-test or the Mann-Whitney-Wilcoxon test. Differential indicators were defined with a false discovery rate (FDR) < 0.05 and an absolute log2(Fold change) > 1. ROC analysis was conducted to evaluate the diagnostic performance of indicators, with AUC values used to select the top 20 diagnostic markers, utilizing the ‘pROC’ package in R. Missing or null values were first identified and imputed using the mean value for each test item in R. If an entire column contained null values, it was removed from the analysis. Graphical visualization was carried out using the ‘ggplot2′ package in R, with further refinements in color and layout made in Adobe Illustrator, as described in prior works (Chang et al., 2021; Chang et al., 2023; Chang et al., 2025).

## Results

### Temporal trends and distribution patterns of stone cases from 2013 to 2023

**FIGURE 1:**
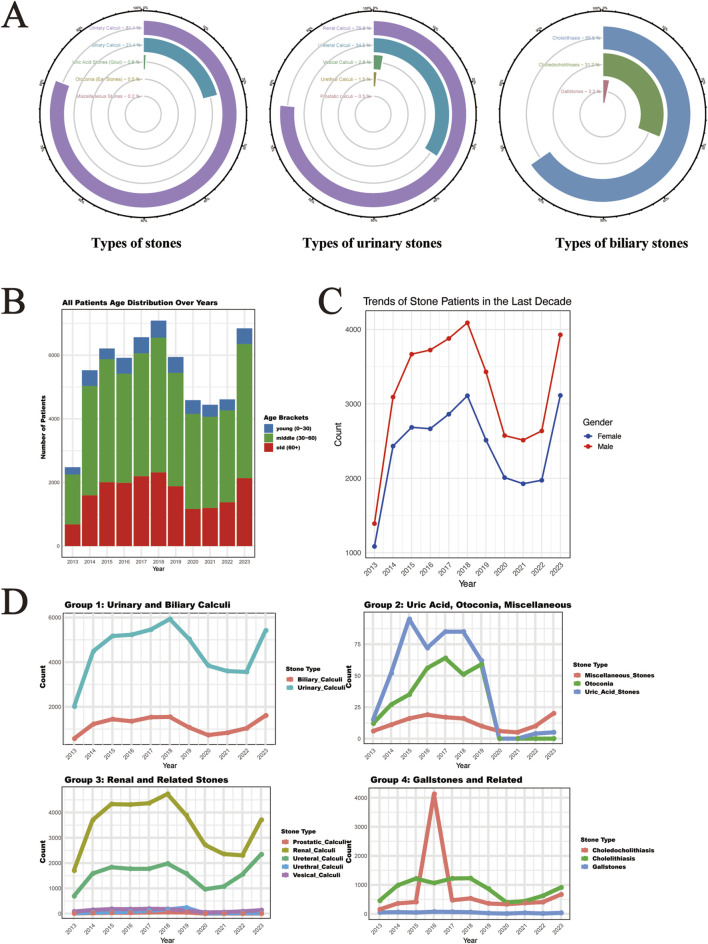
Time Trends and Distribution Patterns of Stone Cases from 2013 to 2023. **(A)** The circular bar chart shows the distribution of stone types, including five major stone categories, five subcategories of urological stones, and the biliary stone system subcategories, representing the overall distribution of 61,310 stone cases. **(B)** The distribution of the ages of stone patients in the last decade. **(C)** The distribution of gender among stone patients in the last decade. **(D)** The time trends of major and subcategories of stones over the last decade (the x-axis represents time, and the y-axis represents the number of cases).

In our comprehensive analysis of 61,310 stone disease cases diagnosed between 2013 and 2023, we observed distinct epidemiological patterns across different organ systems, with an accelerating upward trend in both overall case numbers and specific stone types. Urinary system stones constituted the largest proportion at 80.97% (49,643 cases), followed by biliary system stones at 21.12% (12,951 cases). The remaining cases were distributed among gout (0.77%), otoliths (0.49%), and other stones, including hepatic, testicular, alveolar gastric, submandibular gland, conjunctival, seminal vesicle, spermatic cord, and gastric stones (Figure 1A). Among urinary stones, kidney stones consistently showed the highest incidence, followed by ureteral stones, while in the biliary system, gallbladder stones maintained the highest prevalence, followed by bile duct stones and choledocholithiasis. Demographic analysis revealed a predominance of middle-aged and elderly patients across all systems, with a consistently higher proportion of male patients throughout the study period. This gender disparity and age distribution pattern remained stable across different stone types, though with notable variations in specific subgroups (Figures 1B–D).

Gender distribution analysis revealed significant differences across stone types (Table 1). Among male patients (n = 34,931), urinary calculi were most prevalent (84.06%), with renal calculi (62.48%) and ureteral calculi (30.12%) being the predominant subtypes. In female patients (n = 26,381), while urinary calculi remained the most common (77.22%), there was a notably higher proportion of biliary calculi (24.48% vs. 18.59% in males). Notably, uric acid stones were more common in males (1.11% vs. 0.33%), while otoconia showed female predominance (0.72% vs. 0.33%). These gender-specific differences were statistically significant (P < 0.05) across most stone categories.

**TABLE 1:** Gender distribution of stone patients among 61,312 patients (2013–2023).

Stone style [n/(100%)]	male (n = 34,931)	female (n = 26,381)
Urinary Calculi^a^	29,363 (84.06)	20,371 (77.22)
Renal Calculi	21,825 (62.48)	16,353 (61.99)
Ureteral Calculi^a^	10,521 (30.12)	6620 (25.09)
Vesical Calculi^a^	1,233 (3.53)	157 (0.60)
Urethral Calculi^a^	497 (1.42)	243 (0.92)
Prostatic calculi^a^	234 (0.67)	0
Biliary Calculi^a^	6492 (18.59)	6459 (24.48)
Gallstones^a^	232 (0.66)	238 (0.90)
Cholelithiasis^a^	4860 (13.91)	4531 (17.18)
Choledocholithiasis^a^	2065 (5.91)	2413 (9.15)
Uric Acid Stones^a^	388 (1.11)	87 (0.33)
Otoconia^a^	114 (0.33)	191 (0.72)
Miscellaneous Stones	71 (0.20)	65 (0.25)

^a^ Significantly different between males and females (P < 0.05).

### Construction of matched control cohorts through propensity score analysis

**FIGURE 2:**
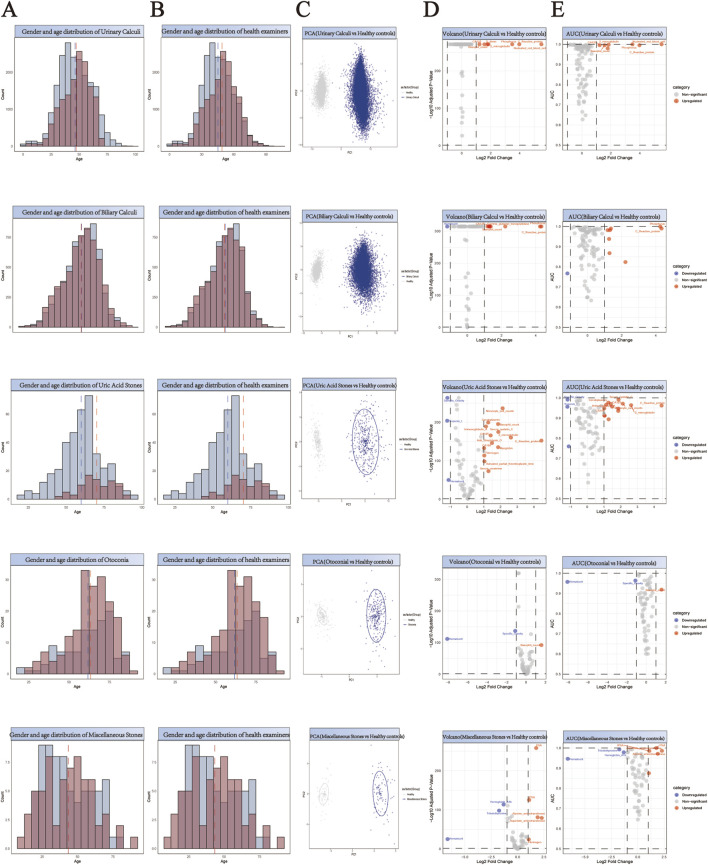
Comprehensive Analysis of Demographics and Biomarker Profiles in Stone Patients *versus* Healthy Controls. **(A,B)** Generate a paired histogram rigorously comparing the age and gender distributions between stone patients and propensity score-matched healthy controls. Blue bars represent males, red bars represent females. The x-axis denotes age, while the y-axis indicates count. Vertical blue and red dashed lines mark the median ages for males and females respectively, demonstrating successful demographic matching between the groups. **(C)** Principal Component Analysis (PCA) plot illustrating the distinct clustering patterns between stone patients (blue) and healthy controls (gray). The X-axis (PC1) represents the first principal component, corresponding to the ​​direction of maximum variance​​ extracted by the PCA algorithm. The Y-axis (PC2) denotes the second principal component, which captures the ​​direction of the second-highest variance​​ orthogonal to PC1. **(D)** Volcano plot analysis of differentially expressed biomarkers. Red points indicate biomarkers significantly upregulated in stone patients compared to controls, while blue points denote downregulated biomarkers. ​​X-axis​​: Log_2_ Fold Change (stone group vs. healthy controls), representing the logarithmically transformed ratio of biomarker expression differences, reflecting their significant overexpression or underexpression in patients. ​​Y-axis​​: Log_10_ Adjusted P-Value, quantifying the statistical significance after multiple testing correction. **(E)** Receiver Operating Characteristic (ROC) curve analysis demonstrates the diagnostic potential of identified biomarkers, with the Area Under the Curve (AUC) value reflecting their discriminatory power in distinguishing stone patients from healthy controls. ​​X-axis (shared with volcano plot)​​: Log_2_ Fold Change (biomarker expression differences between groups), ensuring consistency in fold-change interpretation. ​​Y-axis​​: AUC (Area Under the Curve), quantifying the biomarker’s ability to discriminate between disease and healthy states.

**FIGURE 3:**
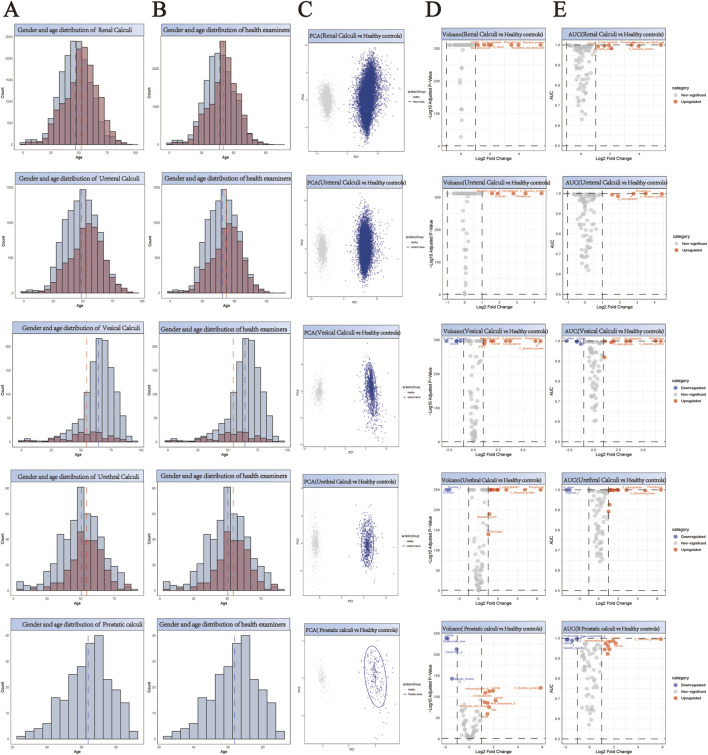
Comprehensive Analysis of Demographics and Biomarker Profiles in Five Subtypes of Urological Stone Patients *versus* Healthy Controls. **(A,B)** Generate a paired histogram rigorously comparing the age and gender distributions between stone patients and propensity score-matched healthy controls. Blue bars represent males, red bars represent females. The x-axis denotes age, while the y-axis indicates count. Vertical blue and red dashed lines mark the median ages for males and females respectively, demonstrating successful demographic matching between the groups. **(C)** Principal Component Analysis (PCA) plot illustrating the distinct clustering patterns between stone patients (blue) and healthy controls (gray). The X-axis (PC1) represents the first principal component, corresponding to the ​​direction of maximum variance​​ extracted by the PCA algorithm. The Y-axis (PC2) denotes the second principal component, which captures the ​​direction of the second-highest variance​​ orthogonal to PC1. **(D)** Volcano plot analysis of differentially expressed biomarkers. Red points indicate biomarkers significantly upregulated in stone patients compared to controls, while blue points denote downregulated biomarkers. ​​X-axis​​: Log_2_ Fold Change (stone group vs. healthy controls), representing the logarithmically transformed ratio of biomarker expression differences, reflecting their significant overexpression or underexpression in patients. ​​Y-axis​​: Log_10_ Adjusted P-Value, quantifying the statistical significance after multiple testing correction. **(E)** Receiver Operating Characteristic (ROC) curve analysis demonstrates the diagnostic potential of identified biomarkers, with the Area Under the Curve (AUC) value reflecting their discriminatory power in distinguishing stone patients from healthy controls. ​​X-axis (shared with volcano plot)​​: Log_2_ Fold Change (biomarker expression differences between groups), ensuring consistency in fold-change interpretation. ​​Y-axis​​: AUC (Area Under the Curve), quantifying the biomarker’s ability to discriminate between disease and healthy states.

**FIGURE 4:**
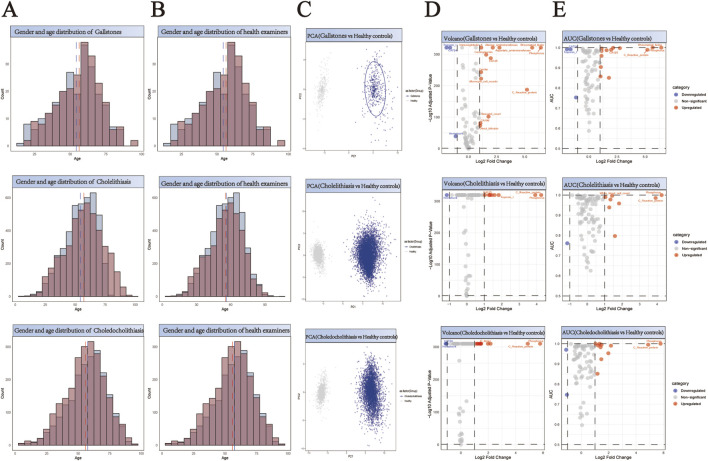
Comprehensive Analysis of Demographics and Biomarker Profiles in Three Subtypes of Biliary Stone Patients *versus* Healthy Controls. **(A,B)** Generate a paired histogram rigorously comparing the age and gender distributions between stone patients and propensity score-matched healthy controls. Blue bars represent males, red bars represent females. The x-axis denotes age, while the y-axis indicates count. Vertical blue and red dashed lines mark the median ages for males and females respectively, demonstrating successful demographic matching between the groups. **(C)** Principal Component Analysis (PCA) plot illustrating the distinct clustering patterns between stone patients (blue) and healthy controls (gray). The X-axis (PC1) represents the first principal component, corresponding to the ​​direction of maximum variance​​ extracted by the PCA algorithm. The Y-axis (PC2) denotes the second principal component, which captures the ​​direction of the second-highest variance​​ orthogonal to PC1. **(D)** Volcano plot analysis of differentially expressed biomarkers. Red points indicate biomarkers significantly upregulated in stone patients compared to controls, while blue points denote downregulated biomarkers. ​​X-axis​​: Log_2_ Fold Change (stone group vs. healthy controls), representing the logarithmically transformed ratio of biomarker expression differences, reflecting their significant overexpression or underexpression in patients. ​​Y-axis​​: Log_10_ Adjusted P-Value, quantifying the statistical significance after multiple testing correction. **(E)** Receiver Operating Characteristic (ROC) curve analysis demonstrates the diagnostic potential of identified biomarkers, with the Area Under the Curve (AUC) value reflecting their discriminatory power in distinguishing stone patients from healthy controls. ​​X-axis (shared with volcano plot)​​: Log_2_ Fold Change (biomarker expression differences between groups), ensuring consistency in fold-change interpretation. ​​Y-axis​​: AUC (Area Under the Curve), quantifying the biomarker’s ability to discriminate between disease and healthy states.

For the purpose of facilitating a comparative analysis of 61,310 stone cases (Supplementary Tables S1-S13), we collected comprehensive health check-up data from 55,010 individuals without any evidence of stone disease (Supplementary Tables S14). A 1:1 propensity score matching strategy was implemented for each stone type to establish balanced control cohorts (Supplementary Tables S15-S27). This matching protocol was specifically designed to minimize demographic bias by ensuring comparable age and gender distributions between stone patients and their respective controls. The effectiveness of our matching approach is demonstrated through paired histograms, where the left panels illustrate the age and gender distributions of stone patients, and the right panels show the corresponding distributions of matched healthy controls (Figure 2A,B; Figure 3A,B; Figure 4A,B). The remarkable concordance in demographic patterns between paired groups validates our matching methodology and establishes a robust foundation for subsequent comparative analyses by effectively controlling for age- and gender-related confounding factors.

## Identification and validation of stone-specific serum biomarkers

Principal Component Analysis (PCA) revealed distinct clustering patterns between stone patients and healthy controls (Figures 2–4C), demonstrating fundamental differences in their biochemical profiles. We constructed volcano plots to identify significantly altered serum biomarkers between stone and control groups using stringent selection criteria (p < 0.05 and log2FC > 0.584962501) (Figures 2–4D). The diagnostic potential of these biomarkers was subsequently evaluated through ROC analysis, employing an AUC threshold of 0.7(Figures 2–4E). Our comprehensive analysis uncovered several stone-specific biomarker signatures. Among the major stone categories, patients with uric acid stones exhibited elevated levels of serum creatinine and serum cystatin C. In urinary system stones, prostatic calculi were characterized by increased PSA and monocyte cell counts. In biliary system stones, the characteristic feature of common bile duct stones is an increase in β2-microglobulin and total bilirubin levels, while gallstones are associated with elevated counts of basophils, ceruloplasmin, ferritin, immunoglobulin-A, and rheumatoid factor levels.

### Biomarker profiling reveals distinct signatures across stone types

**FIGURE 5:**
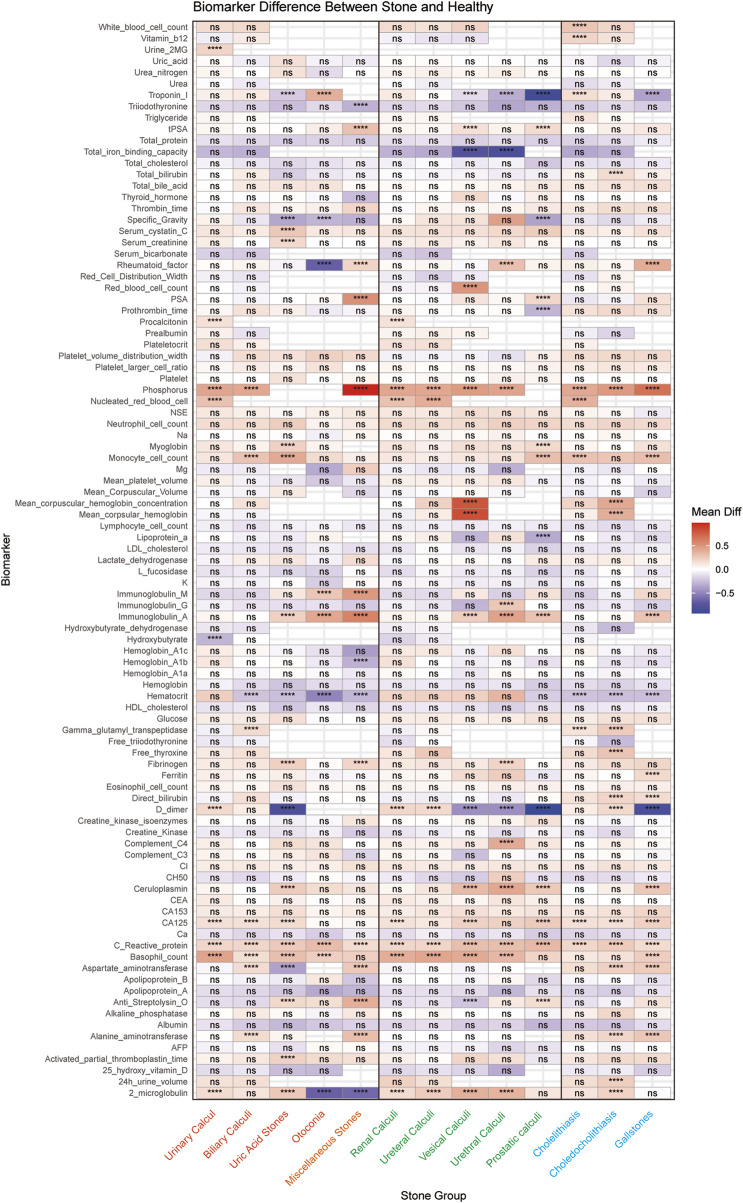
Systematic Comparison of Serum Biomarker Profiles across different Stone Types. The heatmap shows the differential expression patterns of 96 serum biomarkers across different stone types compared to matched healthy controls. The x-axis represents different stone types, and the y-axis shows the biomarkers analyzed. Each cell represents the average difference between the stone group and the control group, with red indicating upregulation and blue indicating downregulation. Statistical significance is indicated by asterisks (* for P < 0.05, ** for P < 0.01, *** for P < 0.001, **** for P < 0.0001), or “ns” for non-significant results. (Orange represents the five major categories of stones, green represents the five subcategories of urological stones, and blue represents the three subcategories of biliary stones).

Through comprehensive biomarker analysis of five major stone categories and their subtypes (five urinary and three biliary), we identified distinct molecular signatures between stone patients and propensity score-matched healthy controls. Statistical evaluation using t-tests and log2FC measurements, coupled with ROC analysis (significance criteria: *FDR*<0.05, log2FC > 0.584962501, AUC>0.7), revealed stone-specific biomarker patterns (Figure 5). Notably, uric acid stones were characterized by elevated levels of ceruloplasmin, activated partial thromboplastin time (aPTT), and myoglobin. Among urinary system stones, prostatic calculi showed increased myoglobin levels with concurrent decreases in prothrombin time, lipoprotein-a, and specific gravity, while urethral calculi exhibited elevations in rheumatoid factor, fibrinogen, and immunoglobulin-G. These distinctive biomarker profiles not only suggest stone-specific pathophysiological mechanisms but also provide potential diagnostic markers for different stone types.

## Discussion

This study represents the first comprehensive evaluation of stone-specific clinical biomarker patterns derived from routine laboratory parameters. We have revealed and validated the elevation of serum creatinine and serum cystatin C levels in patients with uric acid stones. In urological system stones, prostate stones are characterized by increased PSA and monocyte counts. In biliary system stones, common bile duct stones are marked by elevated levels of β2-microglobulin and total bilirubin, while gallstones are associated with increased levels of basophils, ceruloplasmin, ferritin, immunoglobulin-A, and rheumatoid factor.

Cystatin C may be a good marker for GFR, similar to serum creatinine and creatinine clearance, with the advantage of being independent of gender and muscle mass (Randers et al., 1998). In patients with uric acid stones, elevated serum creatinine and cystatin C levels are often associated with kidney damage (Choudhary et al., 2018). Both creatinine-based estimated GFR (eGFRcrea) and serum cystatin C-based estimated GFR (eGFRcys) increase the risk of uric acid stones. Different equations for CysC and/or SCr were compared to estimate GFR (eGFR), offering clues for early detection of kidney function changes in the presence of stones (Liu et al., 2023; Inker et al., 2012). Studies have shown that serum cystatin C is more sensitive than serum creatinine and can reflect kidney function changes earlier, especially in cases of mild kidney damage where serum creatinine levels are not significantly elevated. For example, in some patients with urinary stones, larger kidney stones accompanied by significantly elevated cystatin C levels may indicate early kidney damage (Benoit et al., 2020; Mao et al., 2020). Therefore, serum cystatin C can serve not only as an early marker of kidney health in patients with uric acid stones but also may be more clinically significant than serum creatinine. Incorporate serum creatinine and cystatin C into a dynamic renal function monitoring system. For patients with hyperuricemia, if both markers show persistent elevation (indicating a decline in glomerular filtration rate, GFR), early initiation of 24-h urinary lithogenic component analysis can be implemented. This approach, combined with dual-source CT urate imaging technology, enables non-invasive prediction of stone composition based on metabolic assessment. (Euler et al., 2023).

Prostate stones are a common urological condition, often associated with chronic inflammation seen in histological examinations (De Marzo et al., 2007). While most prostate stones are asymptomatic, larger stones may cause urinary retention or prostatitis through mechanisms that are not yet fully understood (Bedir et al., 2005). Prostate stones have unique characteristics among urinary tract stones, with the most prominent change being the increase in prostate-specific antigen (PSA) levels and monocyte counts (Sindhwani and Wilson, 2005). Studies suggest that prostate stones activate local immune responses, triggering chronic inflammation of the prostate gland and increasing PSA secretion. PSA, a key marker of prostate gland activity, is closely related to the presence of prostate stones. Asymptomatic prostate inflammation has been recognized as an important confounding factor in patients with elevated PSA and is considered a precursor to prostate disease. PSA testing remains the only biomarker for prostate cancer detection and monitoring, but its specificity and sensitivity are limited, often leading to misdiagnosis as prostatitis or prostate stones (Benchikh El Fegoun and Villers, 2007). Notably, there is no clear association between prostate stones and prostate cancer risk (Hwang et al., 2010). Prostate stones are generally categorized into endogenous and exogenous types. Endogenous stones are typically caused by benign prostatic hyperplasia or chronic inflammation, leading to prostate enlargement and subsequent prostate duct obstruction (Anract et al., 2019). Exogenous stones usually form around the urethra, often due to urine reflux into the prostate. Some studies suggest that the formation of prostate stones may be related to inflammatory responses and the deposition of hydroxyapatite crystals in the prostate, with subsequent mineralization involving calcium. In these areas, calcium concentrations may be higher than those at infection sites, triggering chemotaxis responses that lead to the recruitment of inflammatory cells, especially monocytes. An increase in monocytes reflects the immune stimulation of prostate tissue by stones, and the positioning of monocytes and macrophages at injury or inflammation sites is crucial for host defense and extracellular calcium elevation (Olszak et al., 2000; Sugimoto et al., 1993; Wu et al., 2009). It is known that concentrations measured at inflammation sites can induce monocyte chemotaxis, a mechanism that could serve as a potential biomarker for diagnosing prostate stones (Kratofil et al., 2017). Therefore, increased PSA and monocyte counts are common clinical findings in patients with prostate stones, indicating the complex impact of stones on prostate immune responses and helping in the early diagnosis of prostate stones.

In biliary tract stones, one characteristic of common bile duct stones is the elevation of β2-microglobulin (B2M) and total bilirubin levels. This phenomenon is closely related to biliary obstruction, bile stasis, and the associated inflammatory response (Rege and Prystowsky, 1998; Liu et al., 2018). Mechanisms suggest that bilirubin radicals promote the formation of pigment stones in two ways: on one hand, bilirubin radicals have strong aggregation and clumping abilities, which facilitate bilirubin deposition, thus triggering stone formation. On the other hand, bilirubin radicals damage hepatocytes, impairing their function and causing metabolic disturbances, indirectly promoting stone formation (Gillaspie et al., 2019; Liu and Hu, 2002). When the common bile duct is obstructed, bile duct pressure increases, leading to bile reflux and abnormal accumulation of bile components, which in turn raises total bilirubin levels (Gurusamy et al., 2015; Zgheib et al., 2021). At the same time, β2-microglobulin (B2M), as an immune response marker, tends to increase in the infections and inflammation caused by common bile duct stones. B2M is produced by all cells expressing major histocompatibility complex class I antigens, primarily activated lymphocytes (Griffin et al., 2019). In certain disease states, such as infection triggered by common bile duct stones, increased lymphocyte proliferation leads to enhanced B2M synthesis. Studies show that common bile duct stone patients often have elevated total bilirubin and β2-microglobulin levels, which are significant for assessing the severity of biliary obstruction and inflammation caused by the stones. Thus, elevated β2-microglobulin and total bilirubin levels not only reflect obstructive jaundice caused by common bile duct stones but may also serve as indicators of biliary system inflammation and immune response.

In patients with gallstones, the elevation of rheumatoid factor (RF) and basophils may reveal complex immune mechanisms within the biliary system. Elevated RF is typically associated with systemic inflammatory responses or autoimmune diseases, particularly in conditions such as primary biliary cirrhosis, systemic lupus erythematosus, or autoimmune cholangitis. An increase in RF may reflect the presence of chronic biliary inflammation and suggest overactivation of the immune system, potentially exacerbating biliary damage and fibrosis processes (Higashizono et al., 2022). An increase in basophils is commonly linked to allergic reactions, parasitic infections, or immune-mediated chronic inflammation. Biliary obstruction and cholestasis caused by gallstones may activate local immune responses, leading to an increase in basophils. Basophils not only play a significant role in allergic reactions but can also exacerbate biliary inflammation and tissue damage through the release of mediators such as histamine and leukotrienes (Yamanishi and Karasuyama, 2016; Schwartz et al., 2016). Furthermore, the elevation of basophils may be closely related to immune system dysregulation or chronic inflammation triggered by gallstones. Therefore, the increased levels of rheumatoid factor and basophils in gallstone patients not only reflect abnormal immune responses but also provide important clues for assessing the immune functional status of the biliary system and its inflammatory activity, which could serve as a basis for early clinical diagnosis and intervention. Our findings regarding ceruloplasmin, rheumatoid factor, and basophil elevations in gallstone patients suggest novel pathways for diagnostic refinement and therapeutic monitoring. The observed ceruloplasmin elevation may reflect iron-mediated oxidative stress in biliary epithelium. As a key copper transport protein with ferroxidase activity, elevated ceruloplasmin could indicate hepatic iron overload - a known risk factor for pigment stone formation (Kowdley et al., 2019). This finding aligns with recent evidence showing ceruloplasmin upregulation in biliary epithelial cells exposed to lithogenic bile (Banales et al., 2019). The unexpected elevation of rheumatoid factor in gallstone patients challenges conventional understanding of this autoantibody’s specificity. Emerging evidence suggests RF may interact with crystallized cholesterol particles through Fc receptor-mediated mechanisms (Lleo et al., 2012). This immunological cross-reactivity could amplify local inflammation, potentially explaining the association between RF positivity and recurrent cholangitis in our cohort.

While this study provides comprehensive biomarker profiles across stone types, several limitations should be considered. First, the single-center design, though advantageous for standardized laboratory protocols, may limit geographic generalizability given regional variations in stone composition and prevalence. Second, the retrospective nature introduces inherent constraints in establishing causal relationships between biomarkers and stone pathogenesis. Third, while propensity score matching addressed key confounders, unmeasured variables such as dietary patterns or environmental exposures could influence biomarker levels. Fourth, our reliance on serum biomarkers excludes potentially informative urinary or imaging parameters that could enhance diagnostic accuracy. Fifth, the absence of an external validation cohort necessitates caution in extrapolating these findings to other populations. Finally, while we identified promising biomarkers, their clinical utility requires prospective validation in diagnostic algorithms incorporating imaging modalities and clinical parameters. Future multicenter studies incorporating longitudinal designs and multi-omics approaches could address these limitations while exploring therapeutic implications of the identified biomarker patterns.

## Data Availability

The original contributions presented in the study are included in the article/Supplementary Material, further inquiries can be directed to the corresponding authors.
